# A magnetoencephalographic study of longitudinal brain function alterations following carpal tunnel release

**DOI:** 10.1038/s41598-019-56190-8

**Published:** 2019-12-24

**Authors:** Katsuyuki Iwatsuki, Minoru Hoshiyama, Akihito Yoshida, Takaaki Shinohara, Hitoshi Hirata

**Affiliations:** 1 Department of Hand Surgery, Graduate School of Medicine, Nagoya University, Nagoya, Japan; 2 Department of Health Sciences, Faculty of Medicine, Nagoya University, Nagoya, Japan; 3 Brain and Mind Research Center, Nagoya University, Nagoya, Japan; 4 Department of Rehabilitation, Nagoya University Hospital, Nagoya, Japan; 5 Orthopedics Surgery, Daido Hospital, Nagoya, Japan

**Keywords:** Neuropathic pain, Peripheral neuropathies

## Abstract

We investigate changes in brain function before and after carpal tunnel release. Magnetoencephalography (MEG), during which we recorded somatosensory evoked cortical magnetic fields (SEFs), and a clinical evaluation were performed before surgery and 6 months after. The distance on the vertical axis between the equivalent current dipoles (ECDs) for the first and third digits before surgery was significantly less than after surgery. There were no significant differences in values between the control participant and patients after surgery. In terms of distal motor latency, there was a negative correlation with the distance. The recovery function of the root mean square (RMS) before surgery for the N20m was less suppressed at 10 ms of ISI in patients, compared to controls. There were no significant differences in the RMS values for patients before and after surgery. Our results indicate that treating peripheral nerve lesions, such as in carpal tunnel release, positively modifies brain function.

## Introduction

Brain plasticity changes, which can be adaptive or maladaptive, occur following peripheral and central nerve lesions. These changes have been shown in patients with carpal tunnel syndrome (CTS), one of the most common peripheral neuropathies. Research into the plastic changes associated with CTS has primarily used functional magnetic resonance imaging (fMRI)^1–4^ and magnetoencephalography (MEG)^5,6^. Dhond *et al*.^7^ have reported that fMRI and MEG results are novel markers of neuroplasticity in CTS and can be used to study central changes that may occur following clinical interventions.

Both morphological and functional brain changes have revealed brain plasticity. In a study of CTS patients, gray matter volume was significantly reduced in the contralesional somatosensory hand area, pulvinar, and frontal pole. In the contralesional somatosensory cortex, it was correlated with median nerve conduction velocity (NCV)^1^. In addition, cortical thickness in the precentral and postcentral gyri (somatosensory and primary motor hand area) contralateral to the more affected hand was significantly reduced in a CTS-paresthesia subgroup compared with that in a CTS-pain subgroup^3^.

Functionally, a reduced second/third interdigit cortical separation distance in the contralateral primary somatosensory cortex is associated with worse symptomatology (particularly paresthesia), reduced fine motor skill performance, and poorer sensory discrimination accuracy for median nerve innervated digits. Furthermore, primary somatosensory cortex neuroplasticity for median nerve innervated digits in CTS is maladaptive and underlies the functional deficits seen in these patients^2^.

We previously studied patients with CTS using MEG, which revealed functional changes of neural activity in the somatosensory cortex^5^. Distances on the vertical axis between the equivalent current dipoles for the stimulation of the first and third digits were shorter in patients than in controls^5^, in line with a previous report^2^. In addition, the N20m component from the paired median nerve stimulation recovered earlier in CTS patients than in control participants, suggesting that CTS induced disinhibition or hypersensitivity in the primary somatosensory cortex. The local lesion at the peripheral nerve can be treated by carpal tunnel release. We questioned whether the central nervous system responses that had been modified by a peripheral nerve lesion could be remodified by the treatment. Few studies have measured the brain changes in CTS patients before and after treatment. Therefore, the changes in brain plasticity following carpal tunnel release remain unknown. To address this, we conducted a survey 6 months after carpal tunnel release and investigated the changes in brain function before and after treatment.

## Methods

The study was approved by the Ethical Review Committee of Nagoya University. The methods utilized conformed to the applicable regulations and guidelines, and informed consent was obtained from all participants.

### Patients and control participants

The effect of carpal tunnel release was investigated in 12 hands (6 right, 6 left) of 10 patients (7 female, 3 male; 66.8 ± 11.3 years average age). The control group was compromised of 21 age-matched healthy participants (9 female, 12 male; 68.0 ± 7.4 years mean age).

Two individuals in the experimental group suffered from bilateral CTS. As is common practice, both initially underwent carpal tunnel release on the more severe side only. After the conclusion of the investigation of the initial surgery, the condition of their contralateral hand worsened such that it too was operated on. Therefore, the effect of carpal tunnel release was investigated independently on each side in these two patients.

The basis of CTS diagnosis was a history of dysesthesias at points around the median nerve, as reported previously^8^. Nerve conduction studies were performed to confirm the diagnosis. Patients whose CTS symptoms were related to trauma, dialysis, rheumatoid arthritis, or neurological disorders were excluded.

For the clinical assessment, we evaluated grip and pinch strength and administered the Hand10, a patient-rated outcome measure to assess hand disorders, just before and 6 months after surgery^9–12^. Somatosensory evoked cortical magnetic fields (SEFs) were recorded by MEG on the same schedule.

### Magnetoencephalography recording

Signals (0.5 s duration) were recorded using a conventional MEG system as described in our previous study^5^. A 5,000 Hz sampling frequency and an initial bandpass filter (0.3–2,000 Hz range) were employed. Seventy-five channels of signal data from the hemisphere contralateral to the stimulated nerve were used for recovery function and source analysis.

### SEFs following finger stimulation

SEF recordings were taken during stimulation of the first and third digits on the affected hand of CTS patients and on the right hand of control participants. The stimuli were 2 Hz, 0.2 ms square wave pulses randomly delivered to either finger. The intensity was 1.5 times the sensory threshold of each individual^5^.

### Source estimation

To estimate the dipole locations of the initial cortical component of the SEF following stimulation, a single current dipole model was used^13^. Using SEF signals from each hemisphere, equivalent current dipoles (ECDs) with a goodness-of-fit value of 75% were calculated. A three-dimensional plane was used to express the ECD location and calculate the distance between the locations of the two digits on the vertical (z) axis. The midpoint between the pre-auricular points marked the origin of the coordinate system. The x-axis joined the origin to the nasion, with the nasion in the positive direction. The positive y-axis extended from the origin through the left side, and the positive z-axis extended from the origin through the vertex.

### Recovery function

The assessment of the SEF recovery function proceeded as described in previous studies^14–16^. Single or double pulses with interstimulus intervals (ISIs) of 10, 40, 80, or 200 ms were delivered randomly in 0.2 ms durations to the median nerve at the wrist^5^.

To analyze the recovery function, the SEF evoked by the second of the double stimuli was obtained by subtracting the signal obtained from the single stimulation from that of the double stimulation^14–16^. The root mean square (RMS) of the magnetic field obtained from each hemisphere, as a global field power, was calculated. The N20m component was identified within the latency window of 18–24 ms after stimulation.

### Statistical analysis

The distance between the ECD locations was compared using the Tukey-Kramer test. Standardized RMS values were expressed as a function of the ISI. RMS values were also compared in all ISI conditions using the Tukey-Kramer test. Differences were considered statistically significant for p < 0.05.

## Results

### Clinical outcome

All patients showed an improvement in clinical symptoms after the surgery. The mean Hand10 score significantly improved from 33.7 to 13.7. There were no significant differences in grip strength and pinch power before and after treatment (mean grip strength: 21.4 kg before vs. 19.9 kg after; mean pinch power: 6.3 kg before vs. 6.5 kg after). In this analysis, correlations between MEG measurements and Hand10, grip, or pinch strength were not found.

### Source localization of the digits

**Figure 1:**
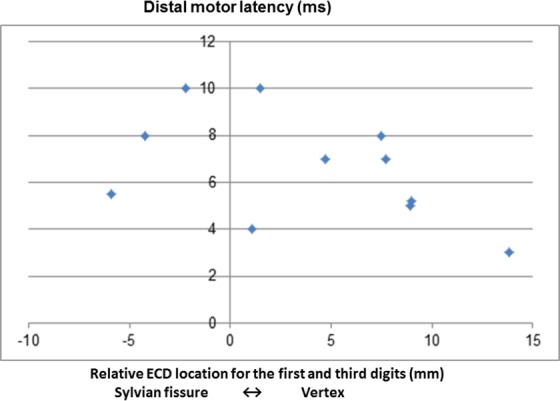
Correlation between distal motor latency and relative equivalent current dipole (EDC) location for the first and third digits. One patient was excluded because distal motor latency was not evoked. (−0.46 correlation coefficient).

To localize the representation of the first and third digits, we calculated ECDs using the N20m elicited on stimulation. In the distance between the first and third digits, there were too many patients in whom sensory nerve conduction could not be detected before the operation, yielding no significant differences. However, in terms of distal motor latency before operation, there was a negative correlation with the conduction distance between the first and third digits (−0.46 correlation coefficient; Fig. 1), suggesting that the conduction distance becomes shorter with increasing severity of CTS^17^.

**Figure 2:**
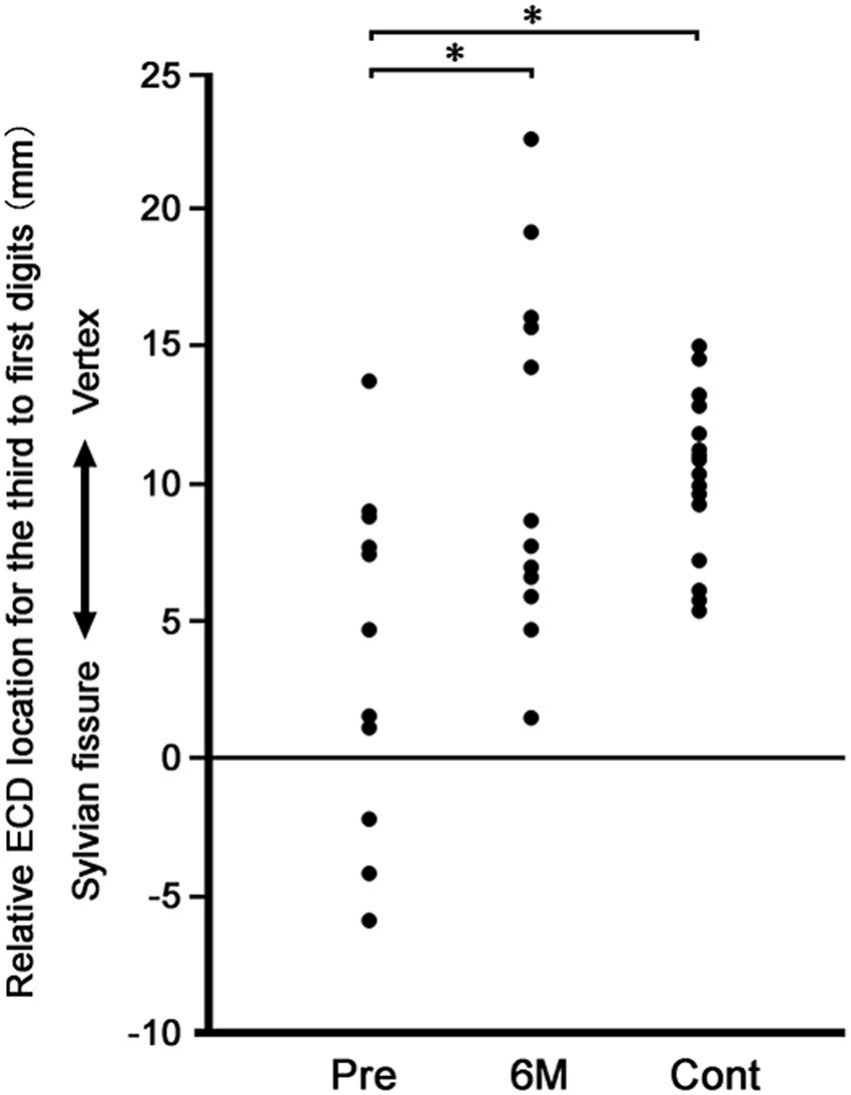
Distance on the vertical axis (z-axis, from the midpoint between pre-auricular points and vertex) between the equivalent current dipoles (ECDs) for the first and third digits in patients before (Pre) and 6 months after (6 M) operative treatment and control participants (Cont). Each dot indicates a value for each participant. Mean values of the distance were smaller in the patients before operation than those after operation and controls (*p < 0.05, Tukey-Kramer test). The zero value indicates the ECD location of the first digit on the z-axis.

The distance on the vertical axis between the ECDs for the first and third digits of patients was significantly greater after surgery, compared to that before surgery. Further, there were no significant differences in this value between the patients after surgery and the control participants (4.1 ± 6.0 cm before, 10.9 ± 6.5 cm after, 11.2 ± 3.1 cm controls; Fig. 2).

### Recovery function

We divided the patients into two groups based on whether sensory nerve conduction was detectable before the operation. In terms of recovery function (10 ms), the mean was 0.46 in the undetectable group (7 hands) and 0.73 in the measurable moderate severity group (5 hands), with the latter being significantly higher (p < 0.05). The mean VAS scores in these two groups were 31.9 and 66.8, respectively (p < 0.05), indicating more severe pain in the moderate severity group.

**Figure 3:**
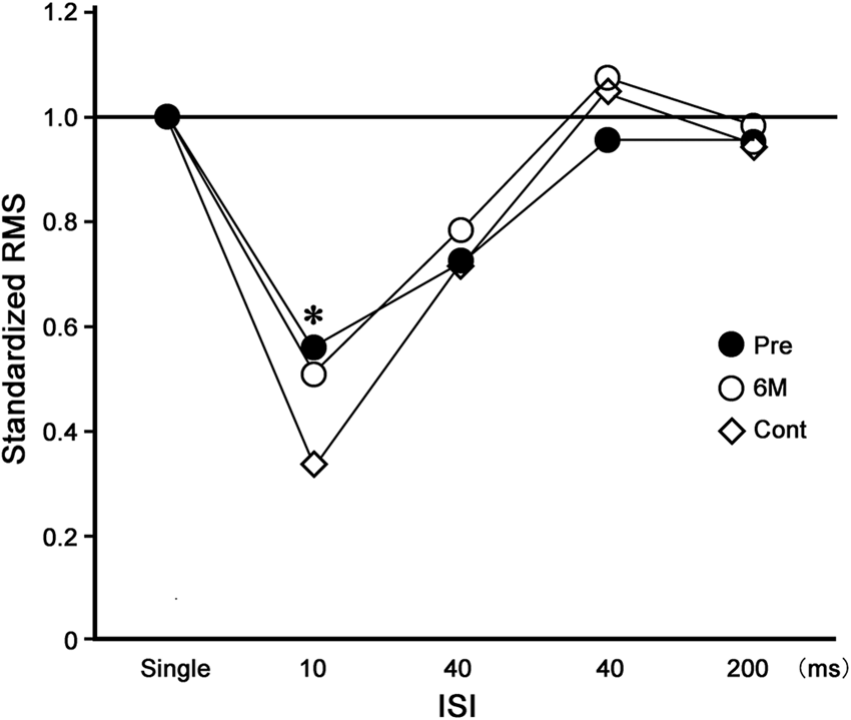
Recovery function of the relative value of the root mean square (RMS) for the N20m following median nerve stimulation at various inter-stimulus intervals (ISIs) on the side operated on in the patients. The RMS value following single stimulation (Single) is expressed as 1.0. The RMSs before (Pre) and 6 months after (6 M) operative treatment in the affected hands and the right hand in the controls (Cont). The mean RMS value at 10 ms of ISI was less suppressed in the Pre condition, compared to the control (*p < 0.05, Tukey-Kramer test).

**Figure 4:**
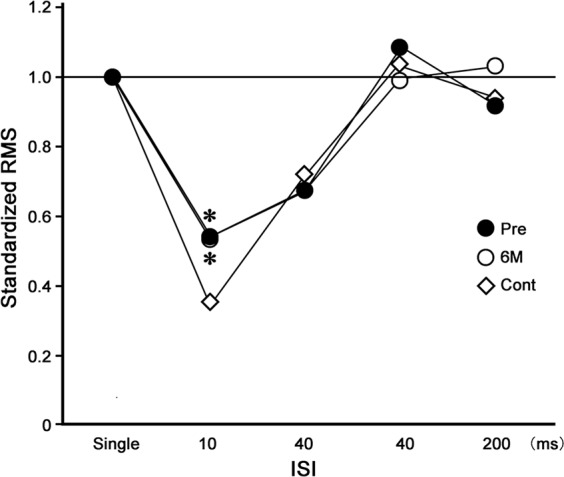
Recovery function of the relative value of the root mean square (RMS) for the N20m following median nerve stimulation at various inter-stimulus intervals (ISIs) on the side not operated on in the patients. The RMS value following single stimulation (Single) is expressed as 1.0. The RMSs before (Pre) and 6 months after (6 M) and the right hand in the controls (Cont). The mean RMS value at 10 ms of ISI was less suppressed in the Pre and 6 M conditions, compared to the control (*p < 0.05, Tukey-Kramer test).

The RMS before surgery for the N20m was less suppressed at 10 ms of ISI in patients, when compared with that in controls (p < 0.05, Figs. 3 and 4). There were no significant differences in the RMS values for patients before and after surgery (Fig. 3). The values were also not significantly different between patients and control participants concerning the hand contralateral to that operated on (Fig. 4).

## Discussion

Many technologies are available to assess brain function including fMRI, positron emission tomography, near-infrared spectroscopy, electroencephalography, and MEG. A particular strength of MEG is that it allows measurement of the magnetic field of the brain caused by the electrical activity of nerves without being affected by extra-cerebral tissues^18^. We previously reported neural function in the brain using a MEG system to elucidate the pathology of intractable pain and nerve disorders^5,16^. CTS causes dysfunction at the peripheral nerve; however, neural signals modified at the peripheral nerve lesion can affect the neural response in the brain^5,19^. In this study, we conducted MEG measurement in patients with CTS before and after surgical treatment to assess any remodeling of neural activity in the brain.

All patients responded well to treatment as evidenced by their Hand10 scores. In comparison to the preoperative distance between ECDs for the first and third digits, the postoperative distance increased, approaching those of health individuals. The increment of the distance between receptive fields for the two fingers indicated that neural activity for spatial discrimination between the fingers after the treatment functionally improved, reaching a level similar to that seen in the controls. On the other hand, the recovery function, which reflects disinhibition or hypersensitivity of the central nervous system following the median nerve stimulation, was not significantly changed by surgical treatment, although a slight shift of the value to the control was observed.

We found two series of neural activities modified by CTS. These plastic changes in the receptive field and recovery function could be interpreted as adaptive or maladaptive. Changes in the receptive fields for the first and third fingers were remodified by 6 months after treatment with improvement of CTS symptoms. The evidence suggests that the change in the receptive fields modified by CTS was maladaptive plasticity in the brain that resulted in poor spatial discrimination between fingers.

The recovery function was not correlated with the clinical symptoms 6 months after surgical treatment. One interpretation might be that the inhibitory/disinhibitory neural activity modified by CTS took longer than 6 months for remodification. Alternatively, disinhibitory neural activity may have been a compensatory adaptive plastic change amplifying neural signals from the peripheral nerve affected by CTS. The disinhibition might have remained until functional repair of the peripheral nerve was completed.

We examined the results of NCV and MEG in this study. Our findings suggested that the conduction distance between the first and third digits might have become shorter with increasing severity of CTS. In other words, the more severe the condition, the more diminished the sensation becomes, which is consistent with the observations in actual clinical practice. More interestingly, there was a larger difference in the recovery function at 10 ms between the moderate severity group and the control group, compared to the difference between the severe group and the control group. This may be because, in the moderate severity group, remodeling in the brain aims to increase the sensitivity through a disinhibitory action on the recovery function and adaptive change; this may be less likely to occur in severe cases, where the patients can no longer feel pain.

Several studies on brain function changes after other orthopedic surgeries have been performed^20–22^. Changes in the brain following contralateral cervical 7 nerve transfer for brachial plexus injury have been reported^21,22^. Furthermore, transhemispheric transposition of activated brain cortices is associated with the recovery of motor and sensory functions in ipsilesional upper limbs in patients with contralateral cervical 7 nerve transfer^21^. Resting-state MRI showed that interhemispheric functional connectivity of the primary motor cortex increased and the inter-hemispheric functional connectivity between the supplementary motor areas reduced bilaterally in patients with CTS^22^. However, these studies reveal brain function after treatment.

Although there are several studies comparing brain function before and after acupuncture treatment for CTS^23–25^, the longitudinal changes associated with surgical treatment of CTS are unknown. Ma *et al*.^6^ used task-dependent fMRI and electromyography assessment before and after surgery to assess CTS changes. They found that CTS patients had decreased activation in the postcentral gyrus, inferior frontal lobe, superior frontal lobe, and parahippocampal gyrus 6 months after surgery. In this study, we used MEG to evaluate plasticity changes before and after carpal tunnel release surgery. We found indexes of brain plasticity change that remained up to 6 months after surgery. To use these MEG and fMRI indexes^6^, we suggest that a new method of treatment evaluation should be developed. Our results show the importance of a neurorehabilitation-based approach to treat not only the diseased site but also the central nervous system. In the future, we plan to evaluate clinical improvement using brain functional analysis.

There are some limitations in this study. First, we had a small number of participants. Similar numbers have been used in previous studies, however, and were considered statistically acceptable. Second, we evaluated changes in plasticity 6 months before and after surgery. This evaluation period matches a previous study^6^; although recovery of function may have been different had we evaluated other periods. Third, the correlation of Hand10 scores, grip strength, and pinch strength with MEG measurements could not be obtained in this analysis. This was not unexpected, as it is well known that clinical data and patient-rated outcome measures do not correlate with electrodiagnostic severity in CTS^26^. Lastly, there is a possibility that the findings might be an epiphenomenon of the surgical intervention. However, because the procedure only involves a small incision with local anesthesia, it is unlikely that any associated epiphenomena might last for the 6 months between our measurement times.

In summary, the distance on the vertical axis between the ECDs for the first and third digits improved 6 months after carpal tunnel release. However, there were only slight changes in the recovery function of the RMS for the N20m 6 months after surgery. Our results suggest that treating peripheral nerve lesions, such as in carpal tunnel release, positively modifies brain function. We reveal a more complete understanding of the pathophysiology of CTS that incorporates a neurorehabilitative approach targeting the adaptive and maladaptive changes in the brain.

## Data Availability

The datasets analysed during the current study are available from the corresponding author on reasonable request.
